# A mutation in *KIF7* is responsible for the autosomal recessive syndrome of macrocephaly, multiple epiphyseal dysplasia and distinctive facial appearance

**DOI:** 10.1186/1750-1172-7-27

**Published:** 2012-05-15

**Authors:** Bassam R Ali, Jennifer L Silhavy, Nadia A Akawi, Joseph G Gleeson, Lihadh Al-Gazali

**Affiliations:** 1 Department of Pathology, Faculty of Medicine and Health Sciences, United Arab Emirates University, Al-Ain, United Arab Emirates; 2 Department of Paediatrics and Neurosciences, University of California, San Diego, USA; 3 Department of Paediatrics, Faculty of Medicine and Health Sciences, United Arab Emirates University, P.O. Box 17666, Al-Ain, United Arab Emirates

**Keywords:** KIF7, Acrocallosal, Joubert, Sonic hedgehog, Dysmorphism, Multiple epiphyseal dysplasia, Fetal hydrolethalus

## Abstract

****Background**:**

We previously reported the existence of a unique autosomal recessive syndrome consisting of macrocephaly, multiple epiphyseal dysplasia and distinctive facial appearance mapping to chromosome 15q26.

****Methods**:**

In this manuscript, we have used whole exome sequencing on two affected members of a consanguineous family with this condition and carried out detailed bioinformatics analysis to elucidate the causative mutation.

****Results**:**

Our analysis resulted in the identification of a homozygous p.N1060S missense mutation in a highly conserved residue in KIF7, a regulator of Hedgehog signaling that has been recently found to be causing Joubert syndrome, fetal hydrolethalus and acrocallosal syndromes. The phenotype in our patients partially overlaps with the phenotypes associated with those syndromes but they also exhibit some distinctive features including multiple epiphyseal dysplasia.

****Conclusions**:**

We report the first missense homozygous disease-causing mutation in *KIF7* and expand the clinical spectrum associated with mutations in this gene to include multiple epiphyseal dysplasia. The missense nature of the mutation might account for the unique presentation in our patients.

## Background

Multiple epiphyseal dysplasia (MED) is a clinical condition characterized by a defect in the process of ossification through mineralization of cartilage. Patients typically present after age two years with joint pain, skeletal abnormalities and short stature. Radiographic investigations point to a generalized delay in epiphyseal ossification together with changes in epiphyseal contour. The range of severity of both clinical and radiographic presentations can be quite striking. Affected joints can include long bones and tubular bones (metacarpals, metatarsals and phalanges), whereas vertebral bodies are only rarely affected [1]. Both dominant and recessive genetic forms exist and can have similar presentations [2].

The *KIF7* gene was first identified as encoding a member of the KIF27 family of kinesin motor domain containing microtubule plus-end directed motors [3]. The KIF27 family in mammals consists of at least 5 members, all with homology to Drosophila Costal-2 protein [3]. All members were found to be implicated in Hedgehog signaling (Hh). However, KIF7 implication in Hh signaling in vertebrates has not been straightforward [4]. Through genetic interaction, *KIF7* gene has been recently shown to be causing Joubert syndrome and the spectrum of fetal hydrolethalus and acrocallosal syndromes [5,6]. Joubert syndrome (JBTS, MIM#213300) is a polygenic condition characterized by a pathognomonic midline “molar tooth” on brain magnetic resonance imaging (MRI) and frequently associated with polydactyly, retinal and renal dysplasia [7]. Fetal hydrolethalus syndrome (HLS, MIM#236680) and acrocallosal syndrome (ACLS, MIM#200990) share features of polydactytly, midline brain and facial abnormalities [8,9]. The implication of KIF7 in these various but phenotypically overlapping conditions suggests a common link with Sonic Hh signaling.

In 1998, we reported an extended consanguineous Omani family with a recessive disorder characterized by distinctive phenotypes including macrocephaly, facial dysmorphism, absent/hypoplastic corpus callosum associated with MED [10], subsequently assigned OMIM entry (%607131). The MED in this family seemed to be distinguishable from other MED syndromes and is part of a larger malformation syndrome, since both craniofacial and central nervous system changes were present. Homzygosity mapping and DNA sequence analysis in this family localized the defective gene close to the telomeric side of the long arm of chromosome 15 (15q26) and resulted in the exclusion of the chondroitin sulphate proteoglycan (*AGC1*) gene [11]. Here we report that the phenotype presented in this family is caused by a homozygous missense mutation in *KIF7*. These findings expand the phenotypic spectrum associated with *KIF7* mutations.

## Materials and methods

### **Clinical description of patients**

**Figure 1:**
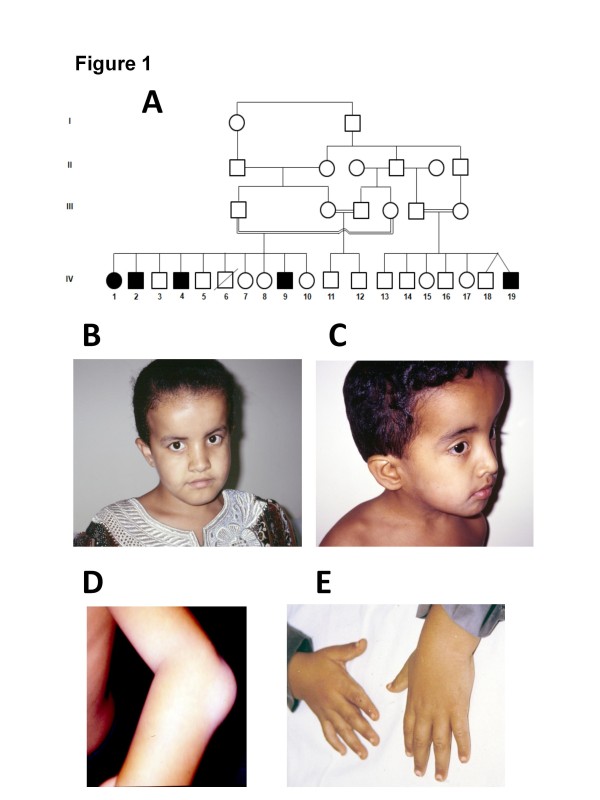
**Pedigree of the affected family with some clinical phenotypes of some members.** (**A**) Pedigree showing consanguinity in all branches of the family. There are four affected members in branch I and one affected in branch III. (**B**) Facial appearance of IV-1 at 12 years of age. Note macrocephaly, frontal bossing with depressed nasal bridge. (**C**) Facial appearance of IV-4. Note macrocephaly, frontal bossing, depressed nasal bridge and low set ears. (**D**) and (**E**) Elbow joint and hands of IV-2. Note prominent elbow joint and wrist joint with spindle shaped fingers.

**Figure 2:**
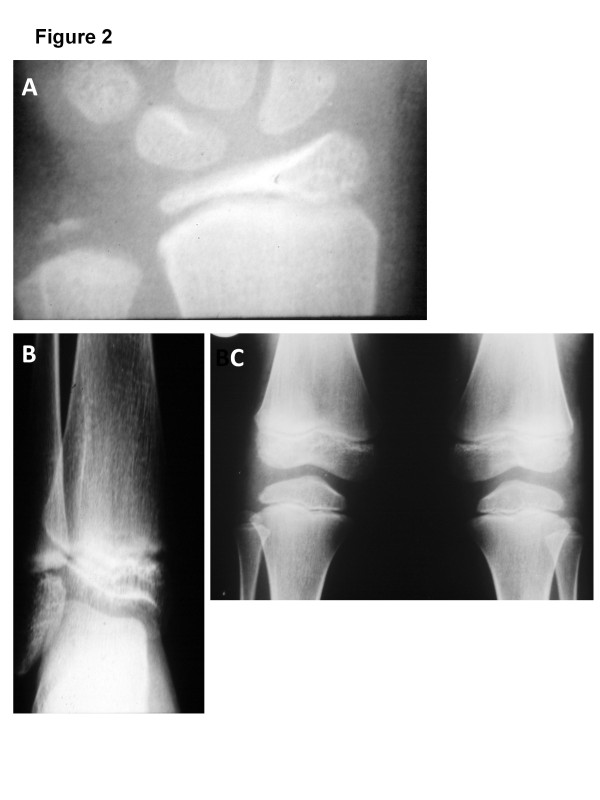
**X-ray images of some affected members. A**) AP film of the wrist in case IV-2 at the age of 10 years showing flattening of the radial epiphyses and underdeveloped ulnar epiphyses. **B**) AP film of the ankle in IV-2 at ten years of age, showing flattening of the tibial epiphyses. **C**) AP film of the knee in case IV-4 at six years of age showing flattening of the tibial and femoral epiphyses with some irregularities of the end plates of femoral epiphyses.

**Figure 3:**
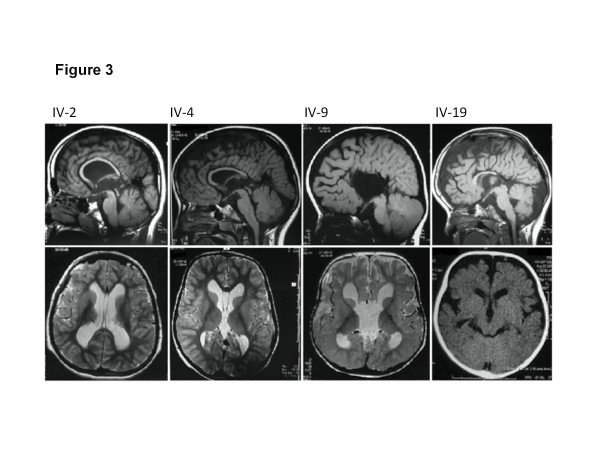
**MRI features in the family.** Identifications corresponding to Figure 1 are indicated above midline sagittal (top) and axial (bottom) images. For IV-19 only axial CT was available. Images show severe thinning of the corpus callosum in IV-2, IV-4 and IV-19. In IV-19 the corpus callosum is probably completely absent. Bottom images show ventriculomegaly (white spaces) and no evidence of a “Molar tooth” sign was evident on brain imaging.

The study was approved by the UCSD and Al-Ain District Human Research Ethics Committees and the family provided informed consent for study. Part of the family has been reported previously [10]. In brief, the family is inbred of Omani origin living in the United Arab Emirates (UAE). There are a total of five children affected in two branches (Figure 1A). All children presented with macrocephaly with frontal bossing, hypertelorism, flat malar regions, low set ears, and short neck (see Figure 1B, C, D and E for examples). In addition, they all have some degree of pectus excavatum, spindle shaped fingers, clinodactyly, prominent joints and genu valgum. Lymphedema was present in two children only. Skeletal survey showed generalized epiphyseal dysplasia in all affected (see Figure 2A, B, and C for examples). Neuroimaging in all except 1 (case 2, not done) showed macrocephaly with hypoplasia of corpus callosum and fronto-temporal brain atrophy (Figure 3). All except 1 had very mild developmental delay (case 2 had normal developmental history) (Table 1).

**Table 1:** **Spectrum of phenotypes of *KIF7 *mutations: literature and present report**

**Features**	**Putoux et al 2011**												**Dafinger et al 2011**				**Al-Gazali and Bakalinova 1997 And this report**				
	F1				F2	F3	F4	F5	F6	F7	F8	F9					IV1	IV2	IV4	IV9	IV19
	P1	P2	P3	P4									P1 (E1)	P2 (E2)	P3 (G1)	P4 (G2)	P1	P2	P3	P4	P5
Brain																					
Macrocephaly	-	-	-	-	-	+	+	+	+	+	-	+	?+	?+	?+	-	+	+	+	+	+
Agenesis/hypoplasia of CC	?	?	+	?	+	+	+	+	+	+	?+	+	+	-	-	-	+	+	+	+	+
Molar-tooth sign	-	-	+	-	+	+	+	+	-	+	+	+	+	+	+	+	+	+	+	+	+
Other brain anomalies	AC	AC	HC, ARC	HC	-	Temporalpachygyria [Abn.hypocampus]	Wide ventricle.	Wide ventricle hypomyel	Wide vent., poor frontal cortical development	-	Para-callosal cyst	-	-	-	-	-	Temporo-parietal atrophy				
Facial																					
Frontal bossing	-	-	-	-	-	-	+	+	+	?+	-	-	+	+	-	-	+	+	+	+	+
Hypertelorism	-	-	-	-	?	?+	+	+	+	+	+	+	+	+	+	-	+	+	?	+	+
Depressed/Wide nasal bridge	-	-	-	-	+	+	+	+	+	+	?	?	?	?	?	?	+	+	+	+	+
Triangular mouth	-	-	-	-	-	-	-	-	-	-	-	-	+	+	-	-	-	+	+	+	+
Hands & Feet																					
Postaxial polydactyly hands	+	-	+	-	-	+	+	-	+	+	-	+	?+	-	-	-	-	-	-	-	-
Postaxial polydactyly feet	+	-	+	-	-	-	+	+	+	+	-	-	-	+	-	-	-	-	-	-	-
Periaxial polydactyly hands	-	-	-	-	-	-	-	-	-	-	-	-	?-	-	-	-	-	-	-	-	-
Periaxial polydactyly feet	+	+	-	+	-	-	-	+	+	-	+	+	-	-	-	-	-	-	-	-	-
Cutaneous syndatyly	-	-	-	-	-	-	-	+	-	-	-	-	-	-	-	-	+ mild webbing	-	-	-	-
Clinodactyly	-	-	-	-	-	-	-	-	-	-	-	-	-	-	-	-	+	+	+	+	+
Spindle shaped fingers	-	-	-	-	-	-	-	-	-	-	-	-	-	-	-	-	+	+	+	+	+
Skeletal																					
Pectus excavatum	-	-	-	-	-	-	-	-	-	-	-	-	-	-	-	-	+	+	+	+	+
Genu valgum	-	-	-	-	-	-	-	-	-	-	-	-	-	-	-	-	+	+	+	+	+
Prominent joints	-	-	-	-	-	-	-	-	-	-	-	-	-	-	-	-	+	+	+	+	+
Epiphyseal dysplasia	-	-	-	-	-	-	-	-	-	-	-	-	-	-	-	-	+	+	+	+	+
Others																					
Mental retardation	NA	NA	NA	NA	+	+	+	+	+	+	+	+	+	+	+	+	V.mild	-	V.mild	V.mild	mild
KIF7 Mutation	Hom c.2896-2897del	Hom c.2896-2897del	Hom c.2896-2897del	Hom c.2896-2897del	Hom R154X	Hom c.1639-1640delinsT	Hom Q1001X	Hom R33X	Hom c.687delG	Hom c.587dupT	Hom c.2896-2897del	Hom c.233-234del	Hom c.217delG	Hom c.217delG	Het c.3986-3997del	Het c.811delG	Hom c.3179A>G	Hom c.3179A>G	Hom c.3179A>G	Hom c.3179A>G	Hom c.3179A>G

*Anc*- anencephaly.

*Hdc*- hydrocephaly.

*Arhc*- arhinocephaly.

*NA*- not applicable.

### **DNA methodologies**

In order to identify the gene for this condition, we performed whole exome sequencing on two affected siblings. Blood DNA was extracted using Qiagen reagents (Qiagen Inc., USA), then subjected to exome capture with the Agilent SureSelect Human All Exome 50 Mb kit (Agilent Technologies, Inc., USA), sequenced on an Illumina HiSeq2000 instrument (Illumina, Inc., USA), resulting in ~94% recovery at >10× coverage. GATK [12] was used for variant identification and intersected with identity-by-descent blocks identified by HomozygosityMapper [13,14], and then filtered for homozygous variants shared between the two affected. In one affected we found 4519 homozygous variants of which 4190 were in the UTRs and therefore probably not functional. In addition, 292 of the remaining 329 variants were seen in homozygous state in families with non-overlapping phenotypes from our in-house 1000 exomes from Middle Eastern patients. Furthermore, 9 of the remaining 38 variants were present in the heterozygous state in 1% or more of the cohort (i.e. out of Hardy Weinberg equilibrium with the disease frequency). Finally, of the remaining 29 variants, only the *KIF7* variant fell within the previously established linkage interval15q26 [11].

## Results and discussion

### **Identification of a missense mutation in the affected family**

**Figure 4:**
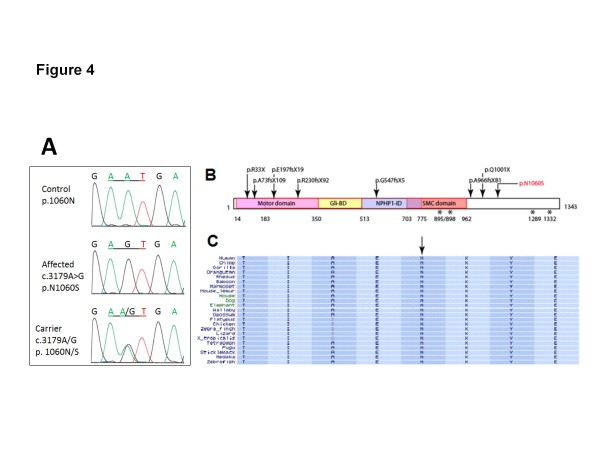
**Identification of a pathogenic mutation in *KIF7 *in the affected family. A**) Sequence chromatogram from control, affected member and a carrier illustrating c.3179A > G mutation. **B**) Depiction of the p.N1060S missense allele (red), as well as all previously identified disease-causing variants (black) along the KIF7 protein domains. The *KIF7* gene encodes a 1343 aa protein with a kinesin motor domain, Gli-binding domain (BD), Nephoronophthisis-1-interacting domain (NPHP1-ID) and a Structural Maintenance of Chromosomes (SMC) domain representing the ATPase activity. Domains are delineated by aa positions below the diagram. Positions 895 and 898 are presumptive phosphorylated sites. **C**) Sequence conservation of the p.N1060 residue across vertebrates.

We identified a mutation at base position chr15:90173657 (according to the hg19 assembly [15], corresponding to a c.3179A > G base transervsion in the *KIF7* gene (accession NM_198525.2). The mutation was found in a homozygous state in all the affected members of this family and is heterozygous in the parents (Figure 4A) and some siblings. It segregated according to a recessive mode of inheritance, as predicted. This mutation was absent from 188 mixed Omani/UAE ethnically matched controls. This single base substitution leads to a change of an asparagine residue in position 1060 of KIF7 to serine (p.N1060S) located after the structural maintenance of chromosomes (SMC) domain of KIF7 (Figure 4B). The asparagine in this position is absolutely conserved in KIF7 family members and related proteins (Figure 4C).

### **The phenotype in the affected family overlaps with acrocallosal syndrome (ACLS)**

The phenotype in acrocallosal syndrome is very broad but Courtens et al. 1997 [16] suggested minimal diagnostic criteria to help confirm clinical diagnosis. Three out of the 4 following criteria should be present: total or partial absence of the corpus callosum, minor craniofacial anomalies, moderate-severe mental retardation and polydactyly. Although the features in our family do not satisfy these criteria, there are many similarities in the phenotype but there are also some major differences. Similarities include corpus callosum anomalies, macrocephaly with frontal bossing and depressed nasal bridge with widely spaced eyes. Polydactyly is considered a major feature of ACLS. All patients reported with ACLS had polydactyly with or without syndactyly (either of hands or feet or both and either preaxial or postaxial) except patient 4 of the family reported by Gelman-Kohan et al. [17]. Polydactyly or syndactyly was absent in all the affected children in our family. One of our cases had mild webbing of the fingers. All the children, however, had spindle shaped fingers which has been reported in some patients with ACLS. Another difference between our family and ACLS is the degree of mental retardation which is usually moderate-severe in ACLS and was very mild or absent in our cases. However, there is 1 report of 2 patients with ACLS with normal development at 7 and 8 years of age [18].

Skeletal abnormalities were major manifestations of the syndrome we reported. These included genu valgum, pectus excavatum and prominent joints [10]. Radiological examination revealed generalized epiphyseal dysplasia in all affected children. Although major skeletal abnormalities were reported in some patients with ACLS syndrome, they are typically restricted to short metacarpals and phalanges [17], osseous defect in parieto-occipital area of the skull, hip dysplasia, bipartite clavicle [8], and metatarsus adductus [16]. MED has not been reported previously in ACLS or other syndromes associated with *KIF7* mutations including hydrolethalis and Joubert syndrome (Table 1).

The presence of MED in a phenotype associated with *KIF7* mutation is not surprising since it has been shown recently that KIF7 has a major role in the growth plate where it regulates hedgehog signaling activity [19]. Chondrocytes proliferation and differentiation in the growth plate requires a proper regulation of the Indian hedgehog (Ihh) signaling which is the major hedgehog ligand in chondrocytes [19]. Ihh signaling in growth plate is mediated by three Gli zinc-finger proteins (Gli1-Gli3) [20]. Studying knockout models indicates that Gli2 and Gli3 are involved in Ihh-dependent chondrocyte development [19]. Mice lacking Ihh do not show ossification in endochondral bones and their chondrocytes exhibit reduced proliferation with an expanded hypertrophic zone in the growth plate [21]. Similarly, *Gli2* knockout mice have an expanded hypertrophic zone and reduced bone formation [22]. This similarity indicated that the Ihh mutant phenotype is partially due to the loss of the Gli2 transcription activation function [22]. On the other hand, loss of Gli3 in the *Ihh* knockout mice reduces the defects in the proliferation and differentiation of chondrocytes [23]. These data demonstrated the importance of Ihh in controlling the transcription repression function of Gli3 in growth plate chondrocytes [23].

**Figure 5:**
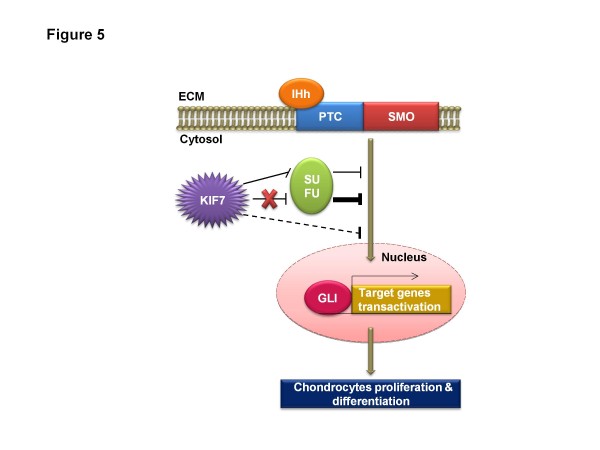
**A simplified scheme to illustrate the dual roles played by KIF7 in Indian Hedgehog (IHh) signaling and chondrocytes development.** In chondrocytes Hedgehog (Hh) signaling pathway is initiated by the IHh ligand binding to PTC, which releases the inhibition of SMO. KIF7 positively modulates IHh pathway activity during development through downregulation of SUFU (indicated by thin straight line). SUFU is a known negative regulator of IHh pathway that prevents GLI-mediated transcription for the genes responsible for chondrocytes proliferation and differentiation during development. In the absence of KIF7, SUFU becomes uninhibited and exerts higher levels of suppression on GLI-mediated transcription (indicated by thick straight line). In the absence of SUFU, KIF7 negatively regulates IHh signaling to a lesser extent by inhibiting GLI-mediated transcription by undefined mechanism (indicated by dashed line). *ECM*: extracellular matrix. *Adapted from Hsu et al.*[19].

Kif7 and another evolutionary conserved protein called Sufu regulate the transcriptional activity of Gli proteins [24]. Hsu et al. [19] demonstrated experimentally that Sufu is a major negative regulator of the Ihh pathway in the growth plate while Kif7 plays dual roles in controlling Ihh signaling and chondrocytes development (Figure 5). They showed that in a wild type chondrocyte, Kif7 positively modulates Ihh pathway activity during development through downregulation of Sufu. These authors showed that in the absence of Sufu, Kif7 negatively regulates Ihh signaling by inhibiting Gli-mediated transcription but the underlying mechanism is not defined. Consistently, Sufu-deficient chondrocytes have augmented Ihh pathway activity, increased proliferation and delayed differentiation while Kif7-deficient chondrocytes exhibited lower Hh pathway activity, a decrease in proliferation and expansion of the hypertrophic zone.

It would be interesting to test *KIF7* gene in other patients with epiphyseal dysplasia and corpus callosum abnormalities in order to further delineate the phenotype of *KIF7* mutations.

## Competing interests

All the authors declare no conflict of interest.

## Authors’ contributions

BRA carried out genetic studies, participated in the sequence analysis and drafted the manuscript. JLS carried out molecular studies, participated in the interpretation and analysis of the whole exome sequencing data and contributed to preparing the manuscript. NAA contributed to data analysis and the writing of the manuscript. JGG and LA recruited and phenotyped the patients, contributed to the analysis of data, supervised the project and prepared the manuscript. All authors read and approved the final manuscript.
